# Speech Kinematics and Coordination Measured With an MEG-Compatible Speech Tracking System

**DOI:** 10.3389/fneur.2022.828237

**Published:** 2022-06-28

**Authors:** Ioanna Anastasopoulou, Pascal van Lieshout, Douglas O. Cheyne, Blake W. Johnson

**Affiliations:** ^1^School of Psychological Sciences, Macquarie University, Sydney, NSW, Australia; ^2^Department of Speech-Language Pathology, University of Toronto, Toronto, ON, Canada; ^3^Hospital for Sick Children Research Institute, Toronto, ON, Canada

**Keywords:** magnetoencephalography, speech motor control, speech coordination, speech disorders, speech kinematics, Articulatory Phonology

## Abstract

Articulography and functional neuroimaging are two major tools for studying the neurobiology of speech production. Until recently, however, it has generally not been possible to use both in the same experimental setup because of technical incompatibilities between the two methodologies. Here we describe results from a novel articulography system dubbed Magneto-articulography for the Assessment of Speech Kinematics (MASK), which we used to derive kinematic profiles of oro-facial movements during speech. MASK was used to characterize speech kinematics in two healthy adults, and the results were compared to measurements from a separate participant with a conventional Electromagnetic Articulography (EMA) system. Analyses targeted the gestural landmarks of reiterated utterances /ipa/, /api/ and /pataka/. The results demonstrate that MASK reliably characterizes key kinematic and movement coordination parameters of speech motor control. Since these parameters are intrinsically registered in time with concurrent magnetoencephalographic (MEG) measurements of neuromotor brain activity, this methodology paves the way for innovative cross-disciplinary studies of the neuromotor control of human speech production, speech development, and speech motor disorders.

## Introduction

While it is relatively straightforward to measure the acoustic consequences of speaking with audio recordings, measuring, and characterizing the physical movements (motor behaviors) that produce acoustic speech signals presents some more formidable challenges. These challenges are due to the inaccessible nature of many of the components of the vocal tract, which are completely or largely hidden from direct view within the laryngeal cavity, the pharynx, the nasal cavity, and the oral cavity. One approach is to simply limit measurements to line-of-sight movements of the lips and jaw, which can be readily characterized with optical (video) tracking and facial capture systems with added precision from placement of reflective (1–4) or active (5) markers. When the focus of interest extends to non-line-of-sight movements researchers must turn to techniques capable of imaging within the cavities of the vocal tract. X-ray microbeam imaging with tongue pellets (6) was originally applied to track tongue movements, and the capability to routinely image movements within the oral cavity has subsequently been extended with ultrasound techniques (7). The more recent advent of real time speech MRI (8) extends speech imaging to visualization of deep soft-tissue structures such as the velum, pharyngeal wall, and the larynx [for a brief overview of these methods, see (9)].

Electromagnetic articulography (EMA; also termed electromagnetic midsagittal articulography or EMMA for older versions of this technology) was developed to image within the oral cavity by tracking movements of marker coils placed on the tongue (10). Movement of the markers within an external magnetic field induces a current in the marker coils and provides high temporal and spatial resolution tracking of movements in real time [see also e.g., Gonzalez et al. (11), Sebkhi et al. (12) for a more recent and contrasting approach using permanent magnet markers and external magnetic sensors]. The tracking coils can also be placed on the lips and jaw and hence this technique provides a powerful method for studies with a focus of interest on intra- and inter-articulator coordination during speech production (13). Relative to other speech tracking techniques, EMA provides more access to the oral cavity than optical methods, better spatiotemporal resolution than ultrasound, and the equipment is considerably more accessible for routine speech research than X-ray beam and MRI speech imaging. As a consequence, EMA has become a central and de-facto standard methodology for research in basic speech science (14) and in neurological disorders of speech motor control (15, 16).

Commercial EMA systems, the Carstens AG series (Carstens Medizenelektronik GmbH, Bovenden, Germany), the recently discontinued NDI Wave [NDI, Waterloo, Canada; see (17)] and other speech tracking methodologies have been crucially important in advancing our understanding of normal and pathological speech behaviors at a very detailed level within the vocal tract. At the same time at the level of the brain, functional neuroimaging techniques have strongly advanced our understanding of the neural activities in centers that control speech movements of the vocal tract. At the present time, however, there remains a fundamental mismatch between the detailed kinematic information available from speech tracking and our understanding of how these parameters are represented and implemented by neural systems. This is because neuroimaging scanners are incompatible with conventional speech tracking technologies (with the exception of video tracking): Ferromagnetic components of movement tracking devices cannot be used within the strong magnetic fields of MRI scanners; and conversely, the electromagnetic fields generated by these devices would swamp the magnetic sensors of MEG scanners. As such, neuroimaging and articulographic studies of speech motor control are typically conducted separately, usually by separate teams of investigators, and it remains difficult to reconcile in detail the results obtained from central vs. peripheral studies of speech neuromotor control. As a consequence, the two types of methods have conventionally been developed and applied in quite separate academic and scientific disciplines: Articulography has been the preferred method in speech science, experimental phonology and speech language pathology, while neuroimaging is a preferred technique in neurolinguistics and cognitive neuroscience. Hence neuroimaging studies have not been able to make use of the detailed information about speech movements of the major articulators provided by articulography, relying instead on simple indices like speech onsets that provide only indirect and very limited indications of the precise movement trajectories of individual articulators. Conversely, articulography measurements have no access to information about the neural activities that generate and control speech movements. The neuroimaging and articulographic aspects of speech production have therefore developed to date as separate and largely independent literatures.

Recent advances in our understanding of speech motor control indicate that it would be advantageous to have access to both types of information in studies of speech production. Most notably, a study by Chartier et al. (18) used ultrasound and video recording of speech movements in conjunction with invasive electrocorticography (ECoG) measurements of neural activity in speech motor cortex of human patients prior to surgery for intractable epilepsy. This study reported that speech motor cortex primarily encodes information about kinematic parameters derived from measurements of the speech movements, rather than acoustic or phonemic parameters derivable from the acoustic speech signal.

**Figure 1:**
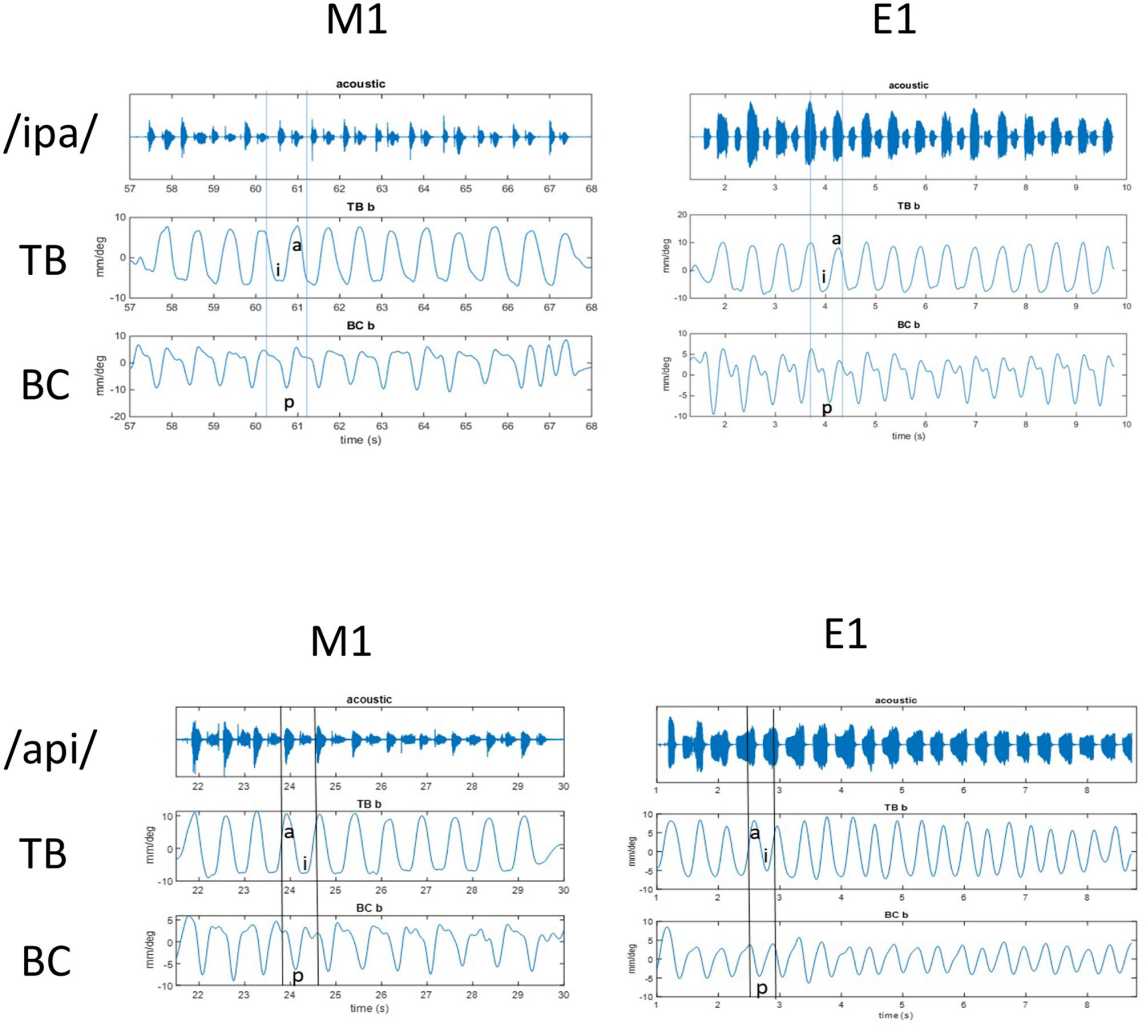
**Top:** Acoustic and kinematic data for repeated [ipa] stimuli at normal rate in MASK (left) and EMA (right). Shown are (from top to bottom) waveform with audio signal, tongue body gesture (TB), and bilabial constriction (BC). Vowel positions are indicated for the 5th reiteration in TB signal and lip closure is indicated for BC signal at same time interval. **Bottom:** Acoustic and kinematic data for repeated [api] stimuli at normal rate in MASK (left) and EMA (right). Shown are (from top to bottom) acoustic signal, tongue body gesture (TB), and bilabial constriction (BC). Vowel positions are indicated for the 4th reiteration in TB signal and lip closure is indicated for BC signal at same time interval.

The recent development of a magnetoencephalograpic (MEG) scanner-compatible speech tracking system (19) finally opens the door for studies that combine high precision measurements of articulator movements with concurrent measurements of the brain activies that control them, at the same time scale and within the same experimental setup. Alves et al. (19) termed the speech tracking system “Magnetoencephalography for the Assessment of Speech Kinematics (MASK).” The MASK system^1^ tracks the independent motion of up to 12 lightweight coils similar in size and shape to the tracking coils used in EMA [see Alves et al. (19), Figure 1]. In an EMA setup, position and orientation of coils are computed from electrical currents passively induced by their movements within a static magnetic field. In contrast, MASK uses active coils energized by sinusoidal currents, whose associated magnetic fields are measured by the MEG sensors. By driving the tracking coils at frequencies greater than about 200 Hz, coil fields can readily be separated by low pass filtering from brain acitivities that are primarily found at frequencies less than about 100 Hz. Coil positions are then localized using the same computational algorithms used in conventional MEG to localize and track head positioning and movement. Importantly, this system does not require line-of-sight tracking, allowing for measurements from all oral articulators including the tongue. As Alves et al. (19) have reported, the MASK system can track articulator movements at rates up to 50 cm/s. The spatial accuracy of MASK is dependent on the distance of the tracking coils from the MEG sensor array. For coils close to the array (e.g., tongue) accuracy is <1 mm relative position error (as with standard MEG head position indicator coils); for coils more distant from the helmet sensor array (e.g., lower lip) spatial accuracy decreases in a non-linear manner to ~1–2 mm.

The current study extends the description of MASK motion tracking capabilities by Alves et al. (19) with a description of MASK capabilities for extraction of higher level kinematic and coordination parameters from the basic movement tracking time series. We aimed to characterize these parameters for speech productions elicited within a standardized reiterated speech production paradigm; to provide a comparison of MASK-derived kinematics with those derived from tracking signals from a conventional EMA system; and to ground the current results from both techniques within the context of the published literature on speech motor control. For the purposes of this “paves the way” special topic issue, we restrict the scope of the current report to a detailed description of MASK-derived kinematics from two participants and will describe the downstream processing of speech-related neuromagnetic data from a larger group of participants in a separate report. Accordingly, the present results pave the way for novel studies of neuromotor control of speech, by providing precise kinematic and coordinative characterization of speech movements, that are intrinsically coregistered in time with MEG measurements of the brain activities that control those movements.

## Methods

### Participants

Three healthy adults with typical developmental histories participated in this study: E1 (F, 30 years, bilingual Hindi/Canadian English, Hindi native speaker); M1 (F, aged 19 years, unilingual native Australian English speaker); M2 (F, 31 years, bilingual Mandarin/Australia English, Mandarin native speaker). E1 participated in the EMA experiment at the Oral Dynamics Lab at the University of Toronto; M1 and M2 participated in the MASK experiment at Macquarie University. All procedures were approved by the University of Toronto and Macquarie University Human Research Ethics Committees.

### Materials

Time-aligned audio and EMA position signals were recorded using the AG501 system (Carstens Medizinelektronik GmbH, Germany) with a large helmet size and automated calibration. EMA coils were attached on the mid-sagittal vermilion border of the upper and lower lip, tongue tip (1 cm from the apex), tongue body (2 cm from the tongue tip), and tongue dorsum (4 cm from the tongue tip) using surgical glue (Periacryl Blue; Gluestitch). Three additional coils were placed at fiducial points on participant's left and right preauricular points and nasion for reference purposes (20, 21)]. After coil attachment, the occlusal bite plane was measured using a custom-made plastic device with two coils attached in the midline at a fixed distance of 3 cm. Before the actual session started, positional information was retrieved to create a standard reference frame (22). Raw movement signals were sampled at 200 Hz and three-dimensional positions over time were calculated from the amplitude recordings (23). The acoustic signal was sampled at 16 KHz. Measurements were carried out with participants in upright seated position.

MASK tracking data and neuromagnetic brain activity were recorded concurrently with a KIT-Macquarie MEG160 (Model PQ1160R-N2, KIT, Kanazawa, Japan) whole-head MEG system consisting of 160 first-order axial gradiometers with a 50-mm baseline (24, 25). MEG data were acquired with analog filter settings as 0.03 Hz high-pass, 1,000 Hz low-pass, 4,000 Hz sampling rate and 16-bit quantization precision. Measurements were carried out with participants in supine position in a magnetically shielded room (Fujihara Co. Ltd., Tokyo, Japan). The occlusal plane and head alignment fiducial points were measured using a hand held digitiser (Polhemus FastTrack; Colchester, VT) and a plastic protractor with three sensors (13). MASK coils were placed at mid-sagittal positions as described above for EMA. Tongue sensors were attached with Epiglu (MajaK Medical Brisbane; Australia), while lip sensors were attached with surgical tape. Participant's head shapes and fiducial positions were digitized (Polhemus FastTrack; Colchester, VT). Marker coil positions affixed to an elastic cap were measured before and after each recording block to quantify participants' head movement, with a maximum displacement criterion of <5 mm in any direction.

Time-aligned speech acoustics were recorded in an auxiliary channel of the MEG setup with the same sample rate as the MEG recordings. An additional speech recording was obtained with an optical microphone (Optoacoustics, Or-Yehuda, Israel) fixed on the MEG dewar at a distance of 20 cm away from the mouth of the speaker; and digitized using a Creative sound blaster X-Fi Titanium HD sound card with 48 kHz sample rate and 8-bit quantization precision. The higher sample rate acoustic recordings were time-aligned off-line with the 4,000 Hz auxiliary speech channel to bring them into time register with the neuromagnetic data.

### Experimental Protocol

Three non- word productions were used as experimental stimuli: Two disyllabic sequences with a V1CV2 structure /ipa/ and /api/; and one trisyllabic sequence /pataka/. The di- and tri-syllabic non-words were selected for measuring intra- (between single articulator movements) and inter- (between consonant and vowel gestures) gestural coordination within a single task (26). The same reiterated stimuli have been used in previous studies investigating speech motor control strategies in normal and in disordered populations (26–29). Non-word stimuli with no linguistic information avoid familiarity issues (29) and have been widely used in the literature to investigate normal and pathological function in speech motor control (30, 31).

Participants were presented with a fixation cross on a display screen and instructed to take a deep breath. The stimulus non-word then appeared on the screen for 12 s. For the normal rate production, participants were required to utter productions at a normal, comfortable rate as they would do while conversing with a friend, until the stimulus non-word disappeared from the screen. For the faster rate, they were instructed to produce the stimuli as fast as possible while maintaining accuracy (28). Following 24, we refer to the reiterated productions generated within the span of a breath intake as a “trial set.” For the EMA session, the subject repeated two trial sets of each production in a randomized order. A short break was provided after each trial set. Participants generated about 15–18 individual productions in each normal rate trial set; and about 20–25 individual productions in each faster rate trial set. Since 100+ individual trials (in this case, individual non-word productions) are typically required for downstream analyses of MEG data, in the MASK sessions the number of trials was increased to 10 trial sets at each rate. For both types of sessions participants were instructed and trained to avoid incorrect speech productions or head movements and they were required to produce each task correctly at the correct rate before data acquisition began.

### Analyses

Magneto-articulography for the Assessment of Speech Kinematics coil position and orientation data initially localized in the MEG sensor frame of reference at a sample rate of 25 Hz was transformed off-line to the occlusal plane and low pass filtered at 6 Hz. These coil locations, orientations, signal magnitudes strength were imported to EGUANA software (9, 21). All tracking data were initially screened for movement artifacts of the acoustic and kinematic signals and subsequent analyses focused on accurate productions (32). /ipa/ and /api/ productions contain a bilabial closure gesture (BC) for the voiceless stop /p/ and two tongue body constriction gestures (TB) for the vowels production /i/ and /a/. The BC gesture was calculated from the two dimensional (x = front-back, y = up-down). Euclidian distance of the upper and lower lip positions and the TB gesture was derived from the two dimensional (x,y) Euclidian distance of the tongue body and the nasion reference coil [see (26)]. /pataka/ contains a bilabial closure (BC) for the voiceless stop /p/, a tongue body (TB) constriction gesture for the vowel /a/, a tongue tip (TT) gesture for the alveolar sound /t/ and a tongue dorsum (TD) gesture for the velar sound /k/. The TT gesture was calculated by the two dimensional (x,y). Euclidian distance of the tongue tip and the nasion reference coil. The TD gesture was calculated by the two dimensional (x,y). Euclidean distance of the tongue dorsum and the nasion reference coil.

Computation of kinematic parameters (amplitude, duration, peak velocity, stiffness, and velocity profile parameter; VPP) were performed for the opening and closing movements of the BC and TB gestures:

- Movement amplitude (with units of mm) refers to the maximum displacement from a peak to a valley and vice versa.
- Movement duration (ms) refers to the time needed for the gesture to move from a peak to a valley and vice versa.
- Peak velocity (mm/s) refers to the maximum velocity achieved by the gesture while moving from a peak to a valley and vice versa.
- Stiffness (1/s) refers to the slope of the relationship between peak velocity and amplitude (33).
- Velocity profile parameter (VPP; arbitrary units), is the stiffness ^*^ duration (34).

Relative phase analysis was used to quantify two types of speech coordination (26, 35):

- Intra-gestural coordination refers to coordination between two individual articulators, in which the coordination of their movement is controlled by the same gesture while.
- Inter-gestural coordination refers to movements controlled by two separate gestures.

As a first step, the power spectra of the BC and TB signals of each trial were computed with the Fourier transform using a frequency resolution of 0.1Hz. The frequency component with greatest power provides a good estimate of the dominant influence on movement patterning over time [(26); see also Namasivayam et al. (36) for more details] and was used as an input for relative phase analysis. A point-differentiation technique was used to derive velocity vs. time from the position signals. The position and velocity signals were then band-pass filtered using the dominant frequency ±0.2 Hz and amplitude normalized. Continuous estimates of relative phase were obtained from the normalized position and velocity functions (28). For intra-gestural coordination, relative phase signals were based on the vertical motion of the upper and lower lip articulators, while for inter-gestural coordination relative phase signals were obtained from gestural data. More specifically, for /ipa/ and /api/ inter-gestural coordination was based on BC vs. TB gestures while for /pataka/ inter-gestural coordination was based on TT vs. BC gestures (for /p/ vs. /t/) and TT vs. TB gestures [for /t/ vs. /k/; see (26)].

## Results

### Raw Tracking Results

The productions /ipa/ and /api/ provide a useful contrast in their mirrored positionings of the tongue and lips and the contrasting positionings are clearly observed in both the MASK and EMA measurements of tongue and lip gestures. In Figure 1 peaks and valleys^2^ indicate the high and low positions achieved by the BC and TB gestures during the production of /api/ and /ipa/. M1 data are from the MASK system and E1 data are from the EMA system. Thus, valleys occur during the bilabial constriction gesture and the tongue body gesture for /i/ and peaks occur for the tongue body gesture of /a/. For /api/, the /p/ closure happens during the upward motion of the TB going from the low /a/ to the high /i/ position. In contrast, for /ipa/, the /p/ closure happens during the downward motion of the TB going from high /i/ to low /a/ position. The gestural movements of /ipa/ and /api/ can be seen as mirror images, where the relative timing of the motions of TB and BC gestures is reversed.

### Kinematic Properties of Individual Speech Gestures

**Figure 2:**
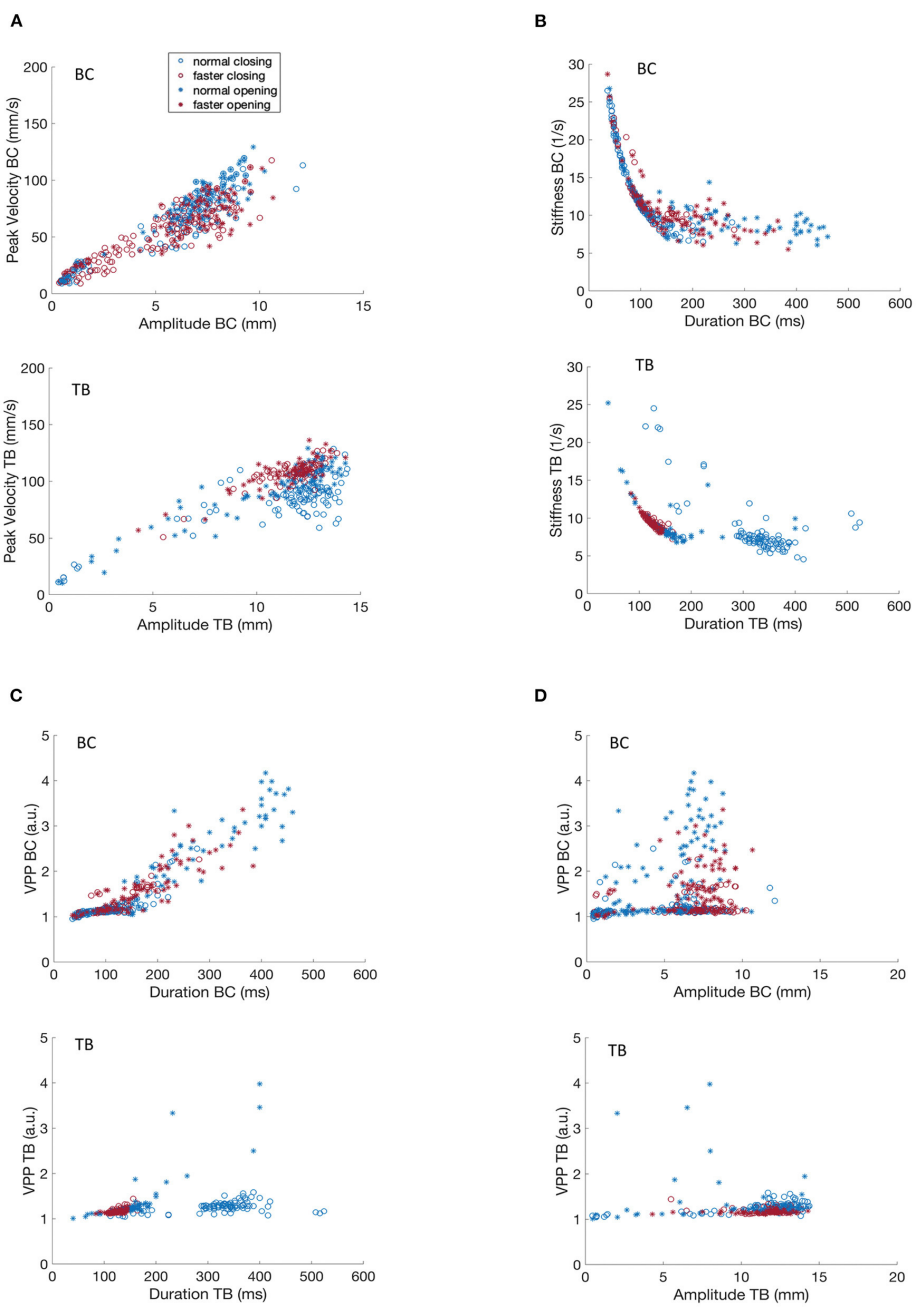
Covariation of kinematic parameters of BC and TB gestures for participant M1 for productions of [ipa]. **(A)** Peak velocity vs. movement amplitude. **(B)** Stiffness vs. duration. **(C)** Velocity profile parameter vs. duration. **(D)** Velocity profile parameter vs. amplitude.

Figure 2 depicts relationships between kinematic parameters (amplitude, duration, and peak velocity) measured for bilabial closure and tongue body gestures during the production of /ipa/ for participants M1 and M2. These data sets are derived from 10 trial sets (each consisting of about 10 productions) for each of normal and faster rates and are shown for both opening and closing movements.

**Figure 3:**
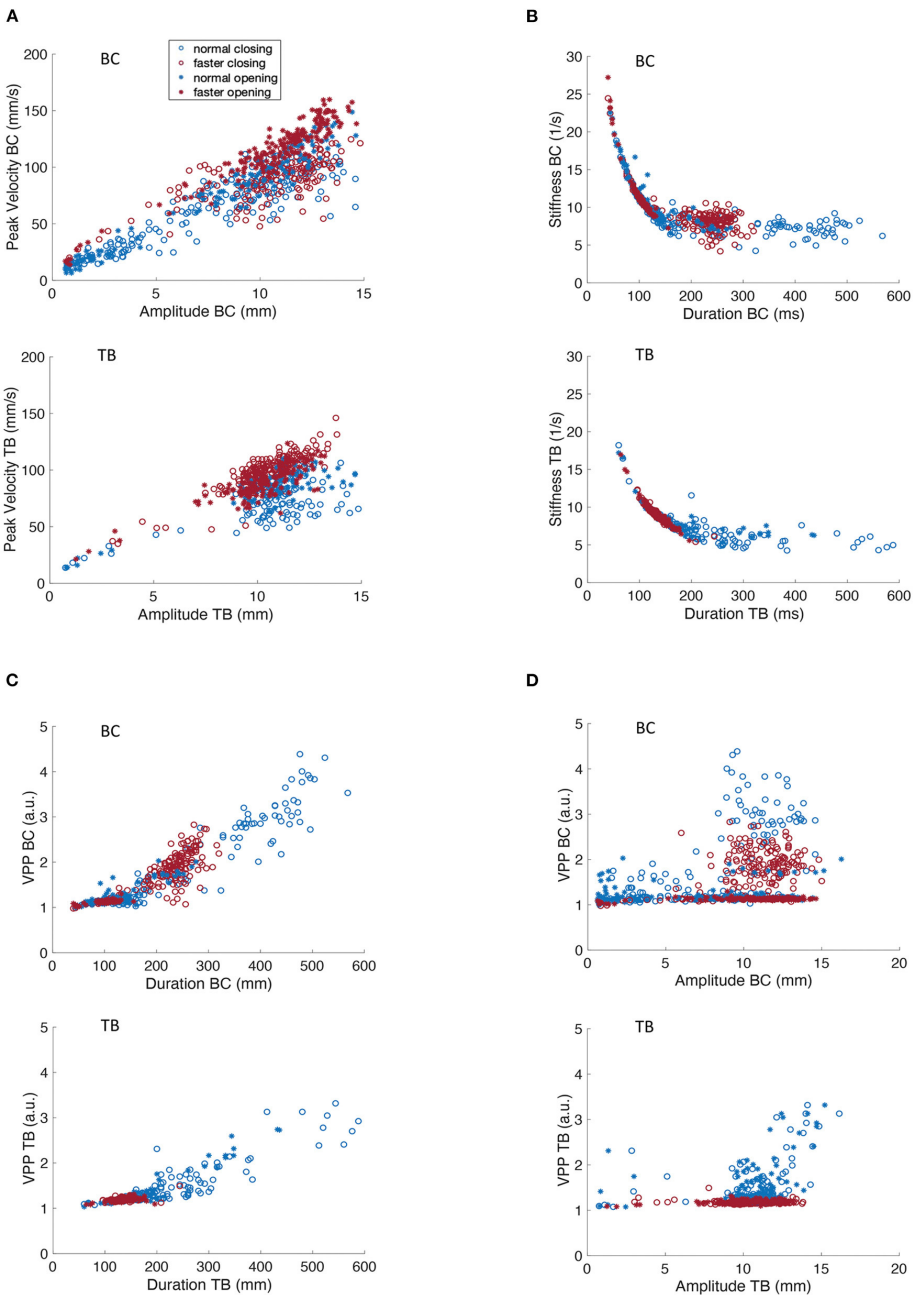
Covariation of kinematic parameters of BC and TB gestures for participant M2 for productions of [ipa]. **(A)** Peak velocity vs. movement amplitude. **(B)** Stiffness vs. duration. **(C)** Velocity profile parameter vs. duration. **(D)** Velocity profile parameter vs. amplitude.

Figures 2, 3A shows that movement peak velocity increased as an overall linear function of movement amplitude, or in other words that greater movement peak speeds are associated with larger movement distances. The clustering of faster rates in the upper right quadrant of both BC and TB plots implies that M1 used a strategy of using greater movement amplitudes at the faster rate. Such speaking strategies can be highly idiosyncratic but in general studies have reported the opposite strategy, i.e., smaller movement amplitudes with faster speaking rates (29, 37). Opening and closing movements show comparable amplitude/velocity relationships indicating that these parameters are controlled in a similar manner regardless of movement direction. This roughly linear covariation of amplitude and peak velocity is a well-known property of speech kinematics and has been well-described for a variety of articulators, gestures and utterances (33, 38).

Figures 2, 3B depicts the covariation of kinematic stiffness with movement duration (STIF = peak velocity/amplitude in units of 1/s) showing that stiffness systematically decreases as a curvilinear function of durations less than about 200 ms while the relationship plateaus into a relatively flat line at greater durations. Clustering of the faster rates is apparent in the lower left quadrant in both BC and TB plots, as faster rates would be expected to have shorter durations. The reason for the greater dispersion of BC data points at the faster rate is unclear, but overall, the plots are entirely comparable to those described previously in the literature (34).

Velocity profile parameter (VPP = STIF ^*^ duration scaled in arbitrary units) is a numerical index of the shape of the velocity profile of a speech movement, whose value varies as a function of the shape of the basis velocity function. As such, velocity profiles have application in motor control both for determining the shape of the potentially underlying control variable (e.g., a purely sinusoidal basis function would have a VPP of pi/2); and for determining if a control parameter pertains or changes across linguistic conditions. Figure 2D shows that TB VPP is essentially constant across the range of amplitudes for opening and closing movements and for normal and faster speaking rates (note that data points for the faster rate are clustered within a narrower range than for normal rate). With greater dispersion of data points, the BC data clearly clusters in a horizontal line centered at a VPP value that is virtually identical to that obtained for TB.

A key contrast in the VPP control regimes for BC and TB is shown in Figures 2, 3C, showing that TB VPP remains constant across durations while BC VPP diverges sharply from the horizontal to a fairly linearly increasing function for durations greater than about 175 ms, indicating that BC, but not TB, systematically scales the velocity control parameter for longer durations. Different velocity control functions at longer durations could be necessitated by the different elastic and hydrostatic properties of the lips and tongue.

In summary, the data of Figures 2, 3 show that kinematic properties derived from speech movements measured with the MASK system demonstrate with high fidelity a number of key kinematic features that have previously been described in the literature. Since these features are highly robust to multiple sources of variance (e.g., rate, gender, developmental age) in human speech they are described as “invariant” properties of speech kinematic movements. Such invariant properties are widely considered to reflect key aspects of motor control of human speech.

### Comparison of Kinematic Features Obtained From MASK and EMA

**Figure 4:**
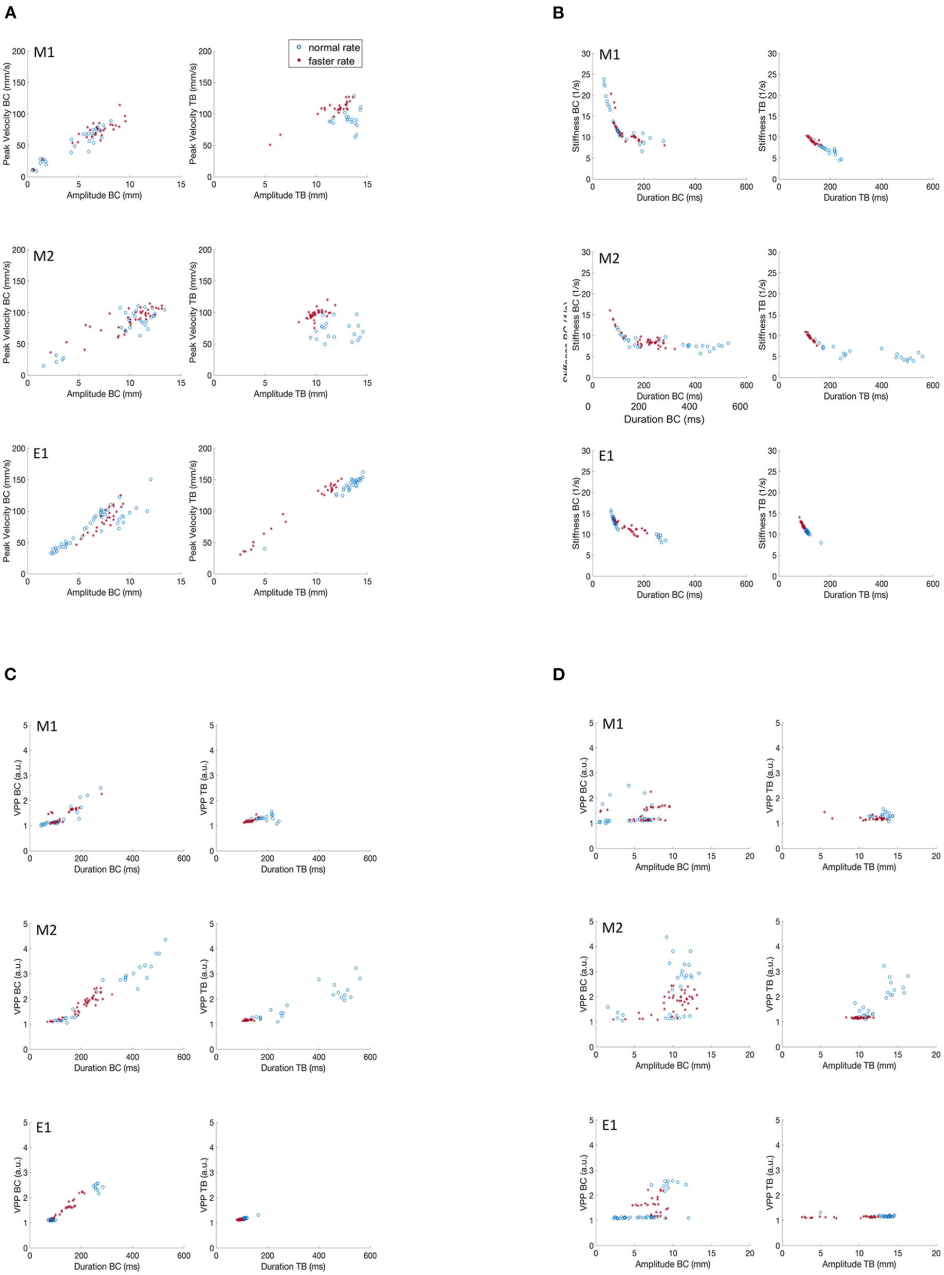
Covariation of kinematic parameters of BC and TB gestures for M1, M2 and E1 for productions of /ipa/. **(A)** Peak velocity vs. movement amplitude. **(B)** Stiffness vs. duration. **(C)** Velocity profile parameter vs. duration. **(D)** Velocity profile parameter vs. amplitude.

Figure 4 recapitulates the kinematic relationships described for subjects M1 and M2 (Figures 2, 3), along with the same data plotted for subject E1. As the EMA session comprised only 2 trial sets, to facilitate comparison we present data only for the first two trial sets for the M1 and M2 participants as well. Even with the lower data sampling, all of the main kinematic features described for M1 and M2 are also evident in the E1 plots: the generally linear increase in peak velocity as a function of movement amplitude (Figure 4A); the curvilinear relationship between stiffness and movement duration (Figure 4C); a generally linear relation between VPP and duration for the BC movement (Figure 4B); and a flat relation between VPP and amplitude, with notably greater dispersion of data points for the BC movement relative to the TB movement. Consistent clustering of faster vs. normal speaker rates are also evident for the TB movements in Figures 4B–D.

In summary, the key kinematic relationships were clearly replicated in the two MASK participants M1 and M2; and both sets of MASK kinematic plots are entirely comparable with those obtained for EMA participant E1. Keeping in mind that the three participants had divergent language backgrounds (Hindi, Mandarin, Australian native English), and that the MASK and EMA experiments were carried out in separate laboratories, these results further support the interpretation that these kinematic profiles reflect relatively stable properties of speech motor control.

### Coordination of Speech Movements

The preceding analyses have focussed on kinematic properties of individual speech movements: we now turn to the matter of coordination of articulator movements within and between speech gestures. In studies of speech motor control coordination is often defined in terms of relative timing as indexed by relative phase between two articulators or two gestures [(26, 28, 39); for alternative conceptualizations of coordination see for e.g., Pearson and Pouw (40) and Vilela Barbosa et al. (41)]. As the present study employed the same stimulus protocol as van Lieshout et al. (26), we adopted their analytic approach in order to provide a direct comparison to their published results. van Lieshout et al. (26) distinguished between “intra-gestural coordination” wherein relative phase signals are based on upper and lower lip movements; and “inter-gestural coordination” where relative phase is calculated from two gestures. TB vs. BC phase coordination was computed for the /ipa/ and /api/ tasks. For /pataka/ TT vs. BC was used to index coordination of tongue and lips movements related to the bilabial and alveolar sound productions /t/ and /p/; while TT vs. TB was used to index phase coordination for the alveolar and velar productions /t/ and /k/.

**Figure 5:**
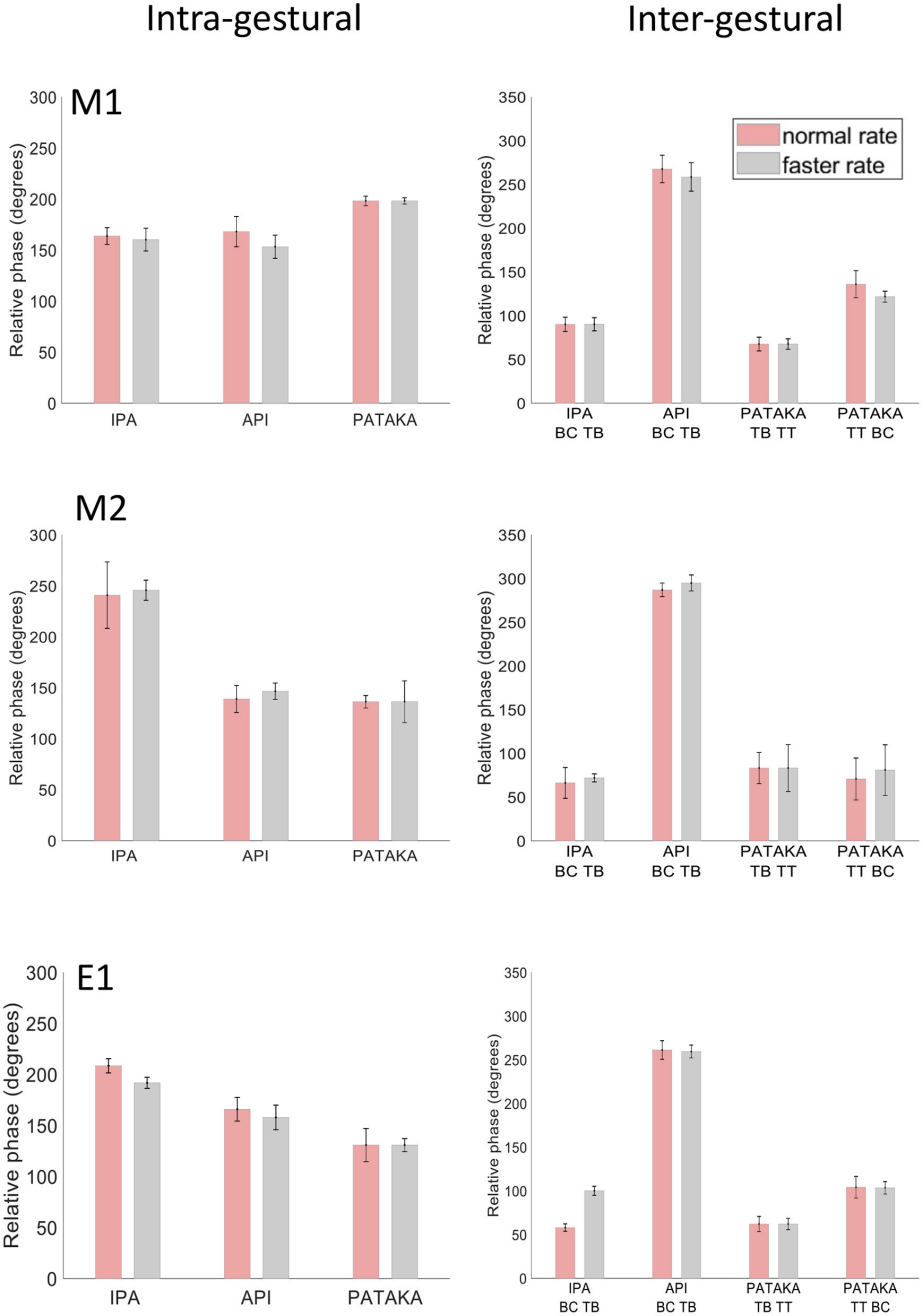
Mean and SD of relative phase for inter- and intra- gestural coordination for participants M1, M2 and E1 at normal and faster rates for productions of [ipa], [api] and [pataka].

We would expect the relative timing of the UL and LL to be consistent across different speaking rates to maintain intelligibility, and indeed intra-gestural coordination of UL and LL in the three speech tasks (Figure 5, left side) showed highly similar relative phase relationships across normal and faster speech rates in all three participants. While the three individuals showed idiosyncratic patterns of intra-gestural coordination across the three speech tasks, the standard deviation bars indicate that the overall variance in intra-gestural coordination was very low for a given production within a given individual. Overall, these results indicate that the relative timing of lip movements is very stable for a given gesture in a specific phonetic context within a given speaker.

As noted above (raw tracking results) the gestural movements of /ipa/ and /api/ can be seen as mirror images, where the relative motions of tongue body and BC gestures are reversed. This reversal is readily apparent in the inter-gestural timing plots of Figure 5 (right side), in all three participants. Notably, the three participants show highly similar relative phase values for individual productions that are preserved across speaking rates, and hence highly comparable patterns of relative phase across the four gestures plotted. These inter-gestural patterns are also entirely comparable to the inter-gestural patterns described for the same gestures by van Lieshout et al. [(26), Figure 9]. Taken together, the present results and the results of van Lieshout et al. (26) indicate that inter-gestural coordination is a highly stable motor control parameter both within and between participants for these types of tasks.

## Discussion

A key characteristic of human speech is that speech goals are consistently achieved in the face of a truly remarkable amount of variation in the conditions under which they must be expressed. The design of the present study captures some aspects of these variable demands: speech movements were measured from participants with widely varying speech acquisition backgrounds, in different laboratories in different countries using different tracking equipment, in upright vs. supine speaking positions, and at different speaking rates. The orderly patterns of speech behaviors that emerge in the present results are thus suggestive of parameters that play key roles in the motor control of human speech.

Such findings conform well to concepts within state speech motor control models such as Articulatory Phonology (AP) and the associated Task Dynamics framework (TD), which hold that articulators create functional relationships to cause local vocal tract constrictions (42). The abstract representations of these articulatory events during speech production are called gestures, the basic units of phonological contrasts (43). Gestures are individual and context-invariant units which can be combined into larger sequences such as syllables, words, and phrases to create meaningful language-specific contrasts. Moreover, gestures are task-specific vocal tract actions which can be implemented by coordinated activity of the articulators in a contextually appropriate manner (44). According to Gafos (45), gestures are described as dynamic spatio-temporal units. In other words, a gesture can be described as “a member of a family of functionally equivalent articulatory movement patterns that are actively controlled with reference to a given speech-relevant goal” (46). A key feature of the AP/TD framework is that gestures can be described within a model of a physical system—the damped mass spring model—with well understood mathematical characteristics.

### Damped Mass Spring Model

In the AP/TD framework, the gestural movements incorporate a specific type of dynamical system, *a point-attractor system* which acts similarly as a dynamical, damped mass-spring-system, i.e., movement of a mass attached to a spring moving toward to an equilibrium position, which produces and releases constrictions of the end-effectors that are being controlled (47, 48). In other words, the starting position of a gesture is analogous to the position of the mass attached to the stretched spring and the equilibrium position is the target position which aimed to be approached by the mass after releasing the spring (47). In this damped mass-spring model there is a specific relationship between the kinematic properties of the gestures: the movement amplitude and movement peak velocity are linearly correlated, and the inverse relationship is observed between the ratio of the peak velocity and amplitude (gestural stiffness) to movement duration. Our results are consistent with the main relationships described in the literature regarding the control system which governs speech gestures (49). More specifically, we found that stiffness increased with longer durations in both BC and TB gestures for all the participants measured with MASK and EMA while the VPP index tends to increase (26, 50). Velocity profiles of normal movements were multipeaked [which indicates a less smooth velocity profile, (51)] while velocity profiles of faster movements were single peaked. The values of VPP decreased in faster rates; values were ~1.57 (π/2) indicating a sinusoidal velocity profile as defined in a frictionless mass-spring model of single axis (51). Moreover, the peak velocity was linearly correlated with movement amplitude thus peak velocity values tend to increase with larger amplitudes; and VPP was not correlated with amplitude as indicated by the straight lines in scatterplots (Figures 2–4).

### Into the Brain

MASK speech tracking data is intrinsically co-registered in time with concurrent MEG measurements of brain activity, providing new capabilities for moving studies of speech motor control from the periphery into the brain. Several recent neuroimaging studies point the direction for how the detailed kinematic and coordinative data described here can be leveraged to address fundamental questions of neuromotor control of speech movements in the human brain. Representational similarity analysis (RSA) (52, 53) is a commonly-used neuroimaging analytic approach which characterizes brain representations in terms of the dissimilarities in brain activity obtained between each pair of experimental conditions in a multivariate experimental design. In their fMRI and MEG study of neuromotor control of hand movements, Kolasinski et al. (54) obtained detailed tracking data of hand movements with a data glove setup as well as electromyographic (EMG) measurements of muscle activity. Using RSA they demonstrated spatially distinct patterns of fMRI activity associated with kinematic and EMG measurements associated with caudal and rostral regions of hand motor cortex respectively; as well as temporally distinct patterns of MEG activity associated with pre-movement and post-movement time windows respectively.

Comparable RSA analyses have been successfully applied to speech movements in several recent fMRI studies. Carey et al. (55) constructed RSA dissimilarity matrices from real-time MRI measurements of laryngeal movements while participants produced steady-state vowels in a speech imitation paradigm. They applied these to fMRI data obtained in a separate session from the real-time MRI session. Their results showed widespread and robust cortical and subcortical activations during per-articulatory sensorimotor transformations during speech imitation. Zhang et al. (56) have extended this approach using theoretical articulatory dissimilarity matrices based on known articulatory dimensions (articulation manner, articulation place, and voicing) of parametrically-varied CV productions; as well as participant-specific acoustic dissimilarity matrices based on acoustic recordings. Their analyses showed that articulatory and acoustic information was represented in distinct and well-defined regions of motor and auditory cortex, respectively. In a recent MEG study, Dash et al. (57) recorded brain signals and jaw motion while participants produced short phrases, and used a decoding model to successfully map brain activity to jaw motion.

The present results show that MASK provides the capability, for the first time, for deriving subject-specific articulatory contrast matrices, based on well-established and robust motor control parameters, in the same experimental setup as the brain recordings and in temporal and spatial co-register with the brain data.

A reviewer of a previous version of this manuscript has noted that it would potentially be of considerable interest to have the capability to record MEG neural activity at a sampling rate comparable to that used in acoustic analyses of speech, i.e., 16 kHz or greater, allowing researchers to probe MEG data for brain activities associated with high frequency acoustic features such as fricatives, in addition to the lower frequency speech movement signals addressed in the present study. This is likely to be possible in the future with ongoing advances in digital storage and processing capacities, but maximal sampling rates of current commercial MEG systems are typically in the range of circa 4 kHz. For the purposes of the present study the acoustic data serve as markers of where events have occurred and the 4 kHz sampling rate is sufficient for lower frequency features such as formants. The time-aligned high sample rate audio signal is used where a more detailed inspection of the acoustic signals is required (e.g., to assess speech errors).

### Implications for Developmental and Clinical Studies

The problem of how humans develop speech is a central, unanswered question of neurolinguistics. The topic has been and remains conspicuously under-studied (58). Studies of this type will inform and constrain theoretical models of language and will have practical implications for significant global medical and health issues. Speech and language problems are the most common and frequent developmental concerns of parents and of speech-language pathologists, general practitioners and pediatricians. These include developmental speech disorders such as stuttering and childhood apraxia of speech; and also, the now well-replicated finding of a greater incidence of comorbid motor coordination and planning problems in children with language impairments (59).

The neural control of speech is also highly relevant to acquired apraxias, and to the burgeoning fields of speech prosthetics and brain computer interfaces (60). It bears on the study of hearing loss, which has profound effects on speech production, and hearing technology including hearing aids and cochlear implants. A greater understanding of the neurophysiology of speech motor control is essential for grappling with the problem that medical interventions can have different effects on speech and non-speech motor control systems: this has been reported for treatments as diverse as levodopa therapy, pallidotomy, fetal, dopamine transplants, and pallidal or thalamic stimulation (61).

## Conclusions

The present results demonstrate that the MASK technique can be used to reliably characterize movement profiles and kinematic parameters that reflect development of speech motor control, while simultaneously measuring the brain activities that provide this control. MASK brings articulography into the brain itself and thereby bridges a crucial methodological gap between the fields of speech science and cognitive neuroscience. The importance of this gap has recently been emphasized by invasive ECoG studies which have demonstrated that speech motor cortex operates by encoding and computing speech kinematic parameters that can be derived only with detailed measurements of the movements of individual articulators, including non-line-of-sight measurements of the oral cavity. This new capability sets the stage for innovative cross-disciplinary efforts to understand the neuromotor control of human speech production.

The impacts of such research flow from the fact that articulography, the current state-of-the-art for studies of speech motor control, measures only the final output of the brain's speech production system. Concurrent MEG neuroimaging powerfully extends the state-of-the-art into the brain itself. In turn, concurrent articulography promises to dramatically improve the precision and inferential power of MEG measures of speech-related brain activity. These studies can therefore facilitate a shift in the current focus of the field and set the stage for new collaborative efforts across a number of disciplines including linguistics, kinesiology, developmental psychology, neuroscience and speech pathology. The results will bear on and eventually inform diagnostic methods and interventions for speech fluency and other motor speech disorders, which are the most common developmental disorders encountered by families, speech-language pathologists, pediatricians, and general practitioners.

## Data Availability Statement

The raw data supporting the conclusions of this article will be made available by the authors, without undue reservation.

## Ethics Statement

The studies involving human participants were reviewed and approved by Macquarie University Human Subjects Ethics Committee and by the Health Science Research Ethics Board at the University of Toronto. The patients/participants provided their written informed consent to participate in this study.

## Author Contributions

IA conducted the experiment. IA and BJ analyzed the data. All authors conceived, designed the experiment, discussed the results, wrote, and edited the manuscript.

## Funding

This work was supported by a Child Development Fund Research Grant from the Waterloo Foundation (Ref. no. 2532 – 4758) and a Discovery Project Grant from the Australian Research Council (DP170102407).

## Conflict of Interest

The authors declare that the research was conducted in the absence of any commercial or financial relationships that could be construed as a potential conflict of interest.

## Publisher's Note

All claims expressed in this article are solely those of the authors and do not necessarily represent those of their affiliated organizations, or those of the publisher, the editors and the reviewers. Any product that may be evaluated in this article, or claim that may be made by its manufacturer, is not guaranteed or endorsed by the publisher.

## Footnotes

^1^The current study used a second generation of the equipment described by Alves et al. (19), with improved electronic design, an increase in number of channels (from 12 to 16), and an increased current output per channel (from 1 to 2 mA) for greater signal amplitude and improved signal to noise ratio.

^2^For TT, TB and TD gestures low position (valley) indicates being closer to palate, and high position (peak) indicates further away from palate. For BC, low position (valley) indicates lips are close together and high position (peak) means lips are further apart. For individual movements, peaks and valleys correspond to the direction for that specific dimension (e.g., peak is up and valley is down for y-dimension).
